# The utility of ^18^F-FDG PET/CT for predicting the pathological response and prognosis to neoadjuvant immunochemotherapy in resectable non-small-cell lung cancer

**DOI:** 10.1186/s40644-024-00772-x

**Published:** 2024-09-10

**Authors:** Rui Guo, Wanpu Yan, Fei Wang, Hua Su, Xiangxi Meng, Qing Xie, Wei Zhao, Zhi Yang, Nan Li

**Affiliations:** 1 Key Laboratory of Carcinogenesis and Translational Research (Ministry of Education/Beijing), Department of Nuclear Medicine, NMPA Key Laboratory for Research and Evaluation of Radiopharmaceuticals (National Medical Products Administration), Peking University Cancer Hospital & Institute, Beijing, China; 2 Key Laboratory of Carcinogenesis and Translational Research (Ministry of Education/Beijing), Department of Thoracic Surgery I, Peking University Cancer Hospital & Institute, No. 52, Fucheng Road, Haidian District, Beijing, 100142 China; 3 State Key Laboratory of Holistic Integrative Management of Gastrointestinal Cancers, Beijing Key Laboratory of Carcinogenesis and Translational Research, Department of Nuclear Medicine, Peking University Cancer Hospital & Institute, Beijing, 100142 China

**Keywords:** FDG, PET/CT, Non-small cell lung cancer, Immunochemotherapy, Neoadjuvant therapy, Pathologic response

## Abstract

**Objective:**

To evaluate the potential utility of ^18^F-FDG PET/CT to assess response to neoadjuvant immunochemotherapy in patients with resectable NSCLC, and the ability to screen patients who may benefit from neoadjuvant immunochemotherapy.

**Methods:**

Fifty one resectable NSCLC (stage IA–IIIB) patients were analyzed, who received two-three cycles neoadjuvant immunochemotherapy.^18^F-FDG PET/CT was carried out at baseline(scan-1) and prior to radical resection(scan-2). SULmax, SULpeak, MTV, TLG, T/N ratio, ΔSULmax%,ΔSULpeak%, ΔMTV%, ΔTLG%,ΔT/N ratio% were calculated. ^18^F-FDG PET/CT responses were classified using PERCIST. We then compared the RECIST 1.1 and PERCIST criteria for response assessment.With surgical pathology of primary lesions as the gold standard, the correlation between metabolic parameters of ^18^F-FDG PET/CT and major pathologic response (MPR) was analyzed. All metabolic parameters were compared to treatment response and correlated to PFS and OS.

**Results:**

In total of fifty one patients, MPR was achieved in 25(49%, 25/51) patients after neoadjuvant therapy. The metabolic parameters of Scan-1 were not correlated with MPR.The degree of pathological regression was negatively correlated with SULmax, SULpeak, MTV, TLG, T/N ratio of scan-2, and the percentage changes of the ΔSULmax%, ΔSULpeak%, ΔMTV%,ΔTLG%,ΔT/N ratio% after neoadjuvant therapy (*p* < 0.05). According to PERCIST, 36 patients (70.6%, 36/51) showed PMR, 12 patients(23.5%, 12/51) had stable metabolic disease(SMD), and 3 patients(5.9%, 3/51) had progressive metabolic disease (PMD). ROC indicated that all of scan-2 metabolic parameters and the percentage changes of metabolic parameters had ability to predict MPR and non-MPR, SULmax and T/N ratio of scan-2 had the best differentiation ability.The accuracy of RECIST 1.1 and PERCIST criteria were no statistical significance(*p* = 0.91). On univariate analysis, ΔMTV% has the highest correlation with PFS.

**Conclusions:**

Metabolic response by ^18^F-FDG PET/CT can predict MPR to neoadjuvant immunochemotherapy in resectable NSCLC. ΔMTV% was significantly correlated with PFS.

**Supplementary Information:**

The online version contains supplementary material available at 10.1186/s40644-024-00772-x.

Lung cancer is the most common cause of cancer death worldwide [1, 2]. More than 70% of patients are already locally advanced at the time of diagnosis. Non-small cell lung cancer(NSCLC) accounts for approximately 80–85% of all lung cancers [3]. Immunotherapy, as a new adjuvant therapy, has shown encouraging efficacy and can restore the function of existing anti-tumor T cells. Chemotherapy enhances anti-tumor immunity through direct or indirect activation of the immune system [4, 5]. Neoadjuvant therapy can reduce tumor burden, increase R0 rate and improve survival rate.In recent years, with the emergence of PD-L1 inhibitors, the strategy of chemotherapy combined with PD-L1 inhibitors has changed the treatment prospects of non-small cell lung cancer [6–8]. The CheckMate 816 study showed that neoadjuvant nivolumab plus chemotherapy significantly improved event-free survival and pathological complete response (pCR) rates compared with chemotherapy alone in patients with resectable NSCLC [7].

Immunotherapy is notoriously expensive, and chemotherapy can cause adverse events such as vomiting and diarrhea in patients [9]. Due to the significant difference between the clinical management strategies of responders and non-responders, there is an urgent need for early assessment of patients who benefit from neoadjuvant immunochemotherapy before or during chemotherapy, and even to predict the treatment response. Finding suitable non-invasive methods is a major problem in clinical practice. ^18^F-fluorodeoxyglucose(FDG)-positron emission tomography/computed tomography (PET/CT) is a useful and non-invasive method to assess tumor size and glucose metabolic status and is widely used to assess tumor stage and treatment response [10–12]. It is very important to evaluate the effectiveness of neoadjuvant immunochemotherapy in NSCLC patients. Misjudgment may lead to wrong decision of next treatment. In particular, there are inconsistencies between CT response assessment criteria and histopathological response in solid tumors. In a meta-analysis, the predictive value of ^18^F-FDG PET/CT for pathological response after neoadjuvant therapy was significantly higher than that of CT scan in NSCLC patients [13]. In addition, ^18^F-FDG PET/CT was considered to provide more useful information for assessing the response to immunotherapy of advanced NSCLC in recent research [1, 14, 15]. At present, there are very few studies on ^18^F-FDG PET/CT in the evaluation of neoadjuvant immunochemotherapy in NSCLC patients. Therefore, the purpose of this study is attempt to evaluate the potential of ^18^F-FDG PET/CT to assess response to neoadjuvant immunochemotherapy in patients with resectable NSCLC.

## Materials and methods

### Patients

We retrospectively reviewed NSCLC patients underwent ^18^F-FDG PET/CT at baseline(scan-1) and prior to surgery(scan-2) from January 2019 to June 2022 at our hospital. The inclusion criteria included the following: (1) Patients had histologically or cytologically confirmed NSCLC (stage IA–IIIB, AJCC 8th), with negative driver mutations(EGFR mutation and ALK rearrangement); (2) received 2–3 cycles of intravenous toripalimab (240 mg) or pembrolizumabon(200 mg) IV q 2-3wks added to nab-paclitaxel (100 mg/m^2^) IV q 2-3wks or pemetrexed (500 mg/m^2^) IV q 2-3wks plus cisplatin 75mg/m^2^ IV q 2-3wks before surgical resection 2 wks; (3) neoadjuvant immunochemotherapy was between scan-1 and scan-2 and patients underwent surgery after neoadjuvant therapy. The exclusion criteria included the following: (1) stage IV non-small cell lung cancer; (2) neoadjuvant immunochemotherapy regimen did not meet the inclusion criteria; (3) The patient did not undergo ^18^F-PET/CT imaging before and after neoadjuvant therapy; (4) The participants were ultimately unable to undergo surgery. Finally, 51 resectable NSCLC patients (6 female and 45 male) with a mean age of 59.80 ± 8.57 years (range: 26–74 years) were enrolled in this study. PET responses were classified using PERCIST criteria [16]. Since this study is retrospective, national laws require neither institutional review board approval nor informed consent.

### PET/CT examination

All patients underwent whole-body PET/CT acquisition 60 ± 10 min after injection ^18^F-FDG by 3.7 MBq/kg. Prior to FDG injection, all patients fasted for at least 6 h. In all cases, the serum glucose concentration met the institutional requirement (≤ 120 mg/dL).

PET/CT scans were performed by Siemens Biograph mCT Flow 64 scanner (Siemens, Erlangen, Germany) which covered the length from the top of skull to the mid-thigh. A low-dose CT scan (120 kV, 35 mA, slice 3 mm) was first performed, and PET acquisition speed was 1.5 mm/s (slice 3 mm, filter: Gaussian, FWHM: 5 mm). A Siemens Biograph mCT Flow 64 scanner PET images were reconstructed using a three-dimensional iterative reconstruction with the time-of-flight algorithm, and the low-dose CT scans were acquired in CARE Dose 4D mode. A Gemini TF scanner PET reconstruction parameters included use of 3D model, and use of ordered-subcohorts expectation maximization(OSEM) method (two iterations, four subcohorts, 128 × 128 pixels of 5.15 mm). Attenuation corrections of the PET images were performed using data from CT imaging.

### Image analysis

A Siemens workstation (Syngo.via VB20, MM Oncology) was used for analyzing all images. Two experienced nuclear medicine practitioners reviewed and analyzed the ^18^F-FDG PET/CT, enhanced CT independently, and any inconsistencies were resolved by consensus.

Volumes of interest (VOIs) were manually drawn for each lesion and the metabolic parameters by lean body mass (SULmax, SULpeak, metabolic tumor volume(MTV), total lesion glycolysis (TLG) and T/N ratio)were automatically calculated. The axial, coronal, and sagittal ^18^F-FDG PET/CT images were qualitatively analyzed by nuclear medicine physicians. The post-treatment percentage changes of metabolic parameters calculated were recorded. In the case of ΔSULpeak%, the formula is as follows: ΔSULpeak% = ( SULpeak of scan-1-SULpeak of scan-2)/SULpeak of scan-1 × 100%. According to PERCIST, response to the neoadjuvant therapy was defined as (1) complete metabolic response (CMR); (2) partial metabolic response (PMR); (3) progressive metabolic disease (PMD); (4) stable metabolic disease (SMD).

The enhanced CT scan was performed before the start of immunochemotherapy and before surgery, respectively. The primary of lung cancer was evaluated with the solid tumor response evaluation standard (RECIST) 1.1. The criteria were as follows: complete response (CR), the lung lesions disappeared; Partial response (PR), the diameter of the lung lesion decreased by at least 30%; Disease progression (PD): the diameter of lung lesions increases by at least 20% or new lesions appear; Disease stability (SD) is neither CR nor PR or PD.

All histological sections were reviewed by two experienced pathologists in accordance with the guidelines recommended by the International Association for the Study of Lung Cancer (IASLC) for pathological evaluation of resected lung cancer specimens after Neoadjuvant Therapy.MPR defined as 10% or less of viable residual tumour, which was assessed by pathologists who measured the percentage of residual viable tumour in resected primary tumours from each patient after surgery.

### Follow-up surveillance

Progression-free survival (PFS) is calculated from the start date of treatment to the date of the first progression (local recurrence or distant metastasis of the tumor) or death from any cause. Overall survival (OS) defined as the time interval from the beginning of induction treatment to death. Evaluate the correlation between metabolic parameters and PFS and OS.

### Statistical analysis

Statistical analysis were conducted by using IBM SPSS (Version 22.0, IBM Corporation, New York, USA). All normal distribution data were tested with Kolmogorov-smirnov and homogeneity of variance with Levene test. SULmax, SULpeak, ΔSULmax%, and ΔSULpeak% were approximately normally distributed, while MTV, TLG, T/N ratio,ΔMTV%,ΔTLG% and ΔT/N ratio% were non-normally distributed. The measurment data were described as means ± standard deviation. Independent sample T tests were used to compare SULmax, SULPeak, and their changes with pathological regression, whereas Mann-whitney U tests were used to compare MTV, TLG, and their changes between the two groups. The correlation between metabolic parameters and MPR in primary tumor after neoadjuvant therapy was evaluated by spearmen’s correlation analysis. The value of metabolic parameters for predicting responders were evaluated by receiver operating characteristic(ROC) curve, and area under the ROC curve(AUC) was calulated. The cutoff was determined using the maximum Youden’s method.Sensitivity, specificity, positive predictive value (PPV), negative predictive value (NPV) and accuracy were calculated. Patients were divided into two categories according to the cutoffs. Kaplan-Meier method and Cox’s Proportional Hazard Model were employed. Statistical significance was set at *P* < 0.05 .

## Results

### Patient characteristics

From January 2019 to June 2022, a total of 51 NSCLC patients(45 males and 6 females, the mean age 59.8 ± 8.6 years old)met the requirements for our study. Among 51 enrolled patients, most of them were male (45/51, 88.2%) ,30 patients received toripalimab added to nab-paclitaxel or pemetrexed plus cisplatin, and 21 patients received pembrolizumabon added to nab-paclitaxel or pemetrexed plus cisplatin. 17 patients(17/51,33.3%) received two cycles of immunochemotherapy, and the rest(34/51,66.7%) received three cycles. Squamous cell carcinoma accounted for 70.6%(36/51), and adenocarcinoma accounted for 25.5%(13/51). The clinical information of the 51 patients were summarized in Table 1.

**Table 1:** Clinical characteristics between patients achieved MPR and Non-MPR

Characteristic	All patients(*n* = 51)	MPR (*n* = 25)	Non-MPR (*n* = 26)
Age (year)	59.8 ± 8.6	62.2 ± 7.1	57.5 ± 10.0
Sex (male/female)	45/6	24/1	21/5
Pathology			
Adenocarcinoma	13	4	9
Squamous cell carcinoma	36	21	15
adenosquamous carcinoma	2	0	2
Clinical stage			
Ia	3	3	0
Ib	8	6	2
IIa	8	3	5
IIb	11	3	8
IIIa	16	6	10
IIIb	5	4	1
Treatment			
Toripalimab + chemotherapy	30	14	16
Pembrolizumab + chemotherapy	21	11	10
History of smoking			
Never	15	9	6
Former or current	36	20	16

### Pathological regression and metabolic parameters

Twenty-five patients(49%, 25/51)pathological regression had achieved MPR, including 21 squamous cell carcinoma and 4 adenocarcinoma, and 18 of these patients achieved pathological complete responses(PCR). Twenty-six patients (51%, 26/51) did not have MPR, but had varying degrees of pathological regression.

Tumour proportion score(TPS), Pathological regression and metabolic parameters of scan-1 and scan-2 after neoadjuvant immunochemotherapy of 51 NSCLC patients were shown in the Supplementary Table S1. There was no statistical significantly between clinical characteristics(gender, age, histology, smoking history, TPS, TNM stage) and MPR. All metabolic parameters of scan-1 had no statistical significant with the degree of pathological regression of lung primary tumor (Table 2). The scan-2 metabolic parameters and the percentage changes of metabolic parameters after neoadjuvant therapy were negatively correlated (*p* < 0.05) with the tumor regression (Table 3).

**Table 2:** The correlation between the metabolic parameters of primary tumor and the degree of pathological regression after neoadjuvant immunochemotherapy

Metabolic parameters	pathological regression	
	*r* value	*p* value
Scan-1		
SULmax	0.012	0.933
SULpeak	0.037	0.795
MTV	0.088	0.539
TLG	0.133	0.351
T/N ratio	0.028	0.845
Scan-2		
SULmax	-0.763	< 0.001
SULpeak	-0.719	< 0.001
MTV	-0.285	0.043
TLG	-0.522	< 0.001
T/N ratio	-0.752	< 0.001
The percentage changes(Δ%) between scan-1 and scan-2		
ΔSULmax%	-0.722	< 0.001
ΔSULpeak%	-0.701	< 0.001
ΔMTV%	-0.394	0.004
ΔTLG%	-0.594	< 0.001
ΔT/N ratio%	-0.717	< 0.001

**Table 3:** Characteristics of ^18^F-FDG PET/CT metabolic parameters with different pathological responses

Metabolic parameters	Tumor with MPR(*n* = 25)	Tumor without MPR(*n* = 26)	*p* value
Scan-1			
SULmax	11.3 ± 4.9	11.7 ± 5.7	0.932
SULpeak	9.9 ± 4.5	9.8 ± 4.9	0.792
MTV	30.3 ± 23.5	40.7 ± 59.1	0.534
TLG	212.7 ± 188.0	2884.7 ± 483.0	0.346
T/N ratio	5.4 ± 2.3	5.5 ± 2.8	0.843
Scan-2			
SULmax	2.1 ± 0.9	9.0 ± 7.1	< 0.001
SULpeak	1.8 ± 1.0	7.2 ± 6.1	< 0.001
MTV	6.7 ± 11.0	11.8 ± 17.4	0.044
TLG	14.1 ± 42.2	88.8 ± 171.3	< 0.001
T/N ratio	0.9 ± 0.4	3.8 ± 3.4	< 0.001
The percentage changes(Δ%) between scan-1 and scan-2			
ΔSULmax%	78.7 ± 11.3	-21.6 ± 42.6	< 0.001
ΔSULpeak%	-78.9 ± 13.1	-25.7 ± 39.7	< 0.001
ΔMTV%	-81.3 ± 19.2	-53.7 ± 45.4	0.005
ΔTLG%	-94.8 ± 6.7	-57.7 ± 42.7	< 0.001
ΔT/N ratio%	-81.0 ± 9.8	-31.0 ± 40.0	< 0.001

The scan-1,scan-2 and the percentage changes of metabolic parameters between MPR and non- MPR were summarized in Table 4. ROC indicated that all of scan-2 metabolic parameters and the percentage changes of metabolic parameters had ability to predict MPR and non-MPR, SULmax and T/N ratio of scan-2 had the best differentiation ability (Table 5). By setting cutoff of SULmax 3.2 and T/N ratio 1.4 of scan-2,the specificity, sensitivity, and accuracy were 84.6%,88.0%,86.3% and 80.8%,88.0% and 84.3%,respectively, with area under curve (AUC) both of 0.94 (*p* < 0.001).

**Table 4:** The potential values of the ^18^F-FDG PET/CT metabolic parameters on predicting MPR

Metabolic parameters	Cut-off	A UC	Sensitivity (%)	Specificity (%)	Accuracy (%)
Scan-2					
SULmax	3.2	0.94	84.6	88.0	86.3
SULpeak	2.8	0.92	76.9	92.0	84.3
MTV	1.9	0.67	84.6	52.0	66.7
TLG	3.0	0.80	92.3	52.0	72.5
T/N ratio	1.4	0.94	80.8	88.0	84.3
The percentage changes(Δ%) between scan-1 and scan-2					
ΔSULmax%	-65.6	0.92	88.5	88.0	88.2
ΔSULpeak%	-68.5	0.91	88.5	88.0	88.2
ΔMTV%	-63.6	0.73	57.7	88.0	72.5
ΔTLG%	-90.9	0.84	69.2	92.0	80.4
ΔT/N ratio%	-68.9	0.91	84.6	88.0	86.3

**Table 5:** The correlation between the clinical information and metabolic parameters of 37 PMR patients and the degree of pathological regression after neoadjuvant immunochemotherapy

Characteristic	Non-MPR (*n* = 12)	MPR (*n* = 25)	*P*
Age (year)	59.58 ± 7.34	62.20 ± 7.11	0.307
Sex (male/female)	8/4	24/1	0.03
TPS	12.33 ± 20.28	18.86 ± 31.27	0.886
Clinical stage	-	-	0.146
Scan-1			
SULmax	11.7 ± 5.8	11.3 ± 4.9	0.911
SULpeak	9.7 ± 4.8	9.9 ± 4.5	0.886
MTV	41.5 ± 67.6	30.3 ± 23.5	0.643
TLG	232.6 ± 356.5	212.7 ± 188.0	0.395
T/N ratio	5.6 ± 2.9	5.4 ± 2.3	0.987
Scan-2			
SULmax	4.4 ± 2.3	2.1 ± 0.9	<0.001
SULpeak	3.3 ± 1.8	1.8 ± 1.0	0.001
MTV	44.2.±67.1	66.5 ± 10.9	0.761
TLG	15.8 ± 29.6	14.1 ± 42.2	0.181
T/N ratio	1.8 ± 1.0	0.9 ± 0.4	<0.001
The percentage changes(Δ%) between scan-1 and scan-2			
ΔSULmax%	-56.6 ± 20.1	78.7 ± 11.3	0.001
ΔSULpeak%	-59.9 ± 19.7	-78.9 ± 13.1	0.003
ΔMTV%	-78.1 ± 24.7	-81.3 ± 19.2	0.886
ΔTLG%	-88.4 ± 13.9	-94.8 ± 6.7	0.077
ΔT/N ratio%	-62.7 ± 18.7	-81.0 ± 9.8	0.002

### Association between metabolic response and pathological response

According to PERCIST, the metabolic response were classified as CMR (0%, 0/51), PMR (72.5%, 37/51), SMD (23.5%, 12/51), and PMD (4.0%, 2/51)( Figrue 1) .A typical case is shown in Figrue 2– 4. Of the 37 PMR patients, 25 achieved MPR and 12 achieved non- MPR after neoadjuvant immunochemotherapy (Figrue 5).

**Fig. 1:**
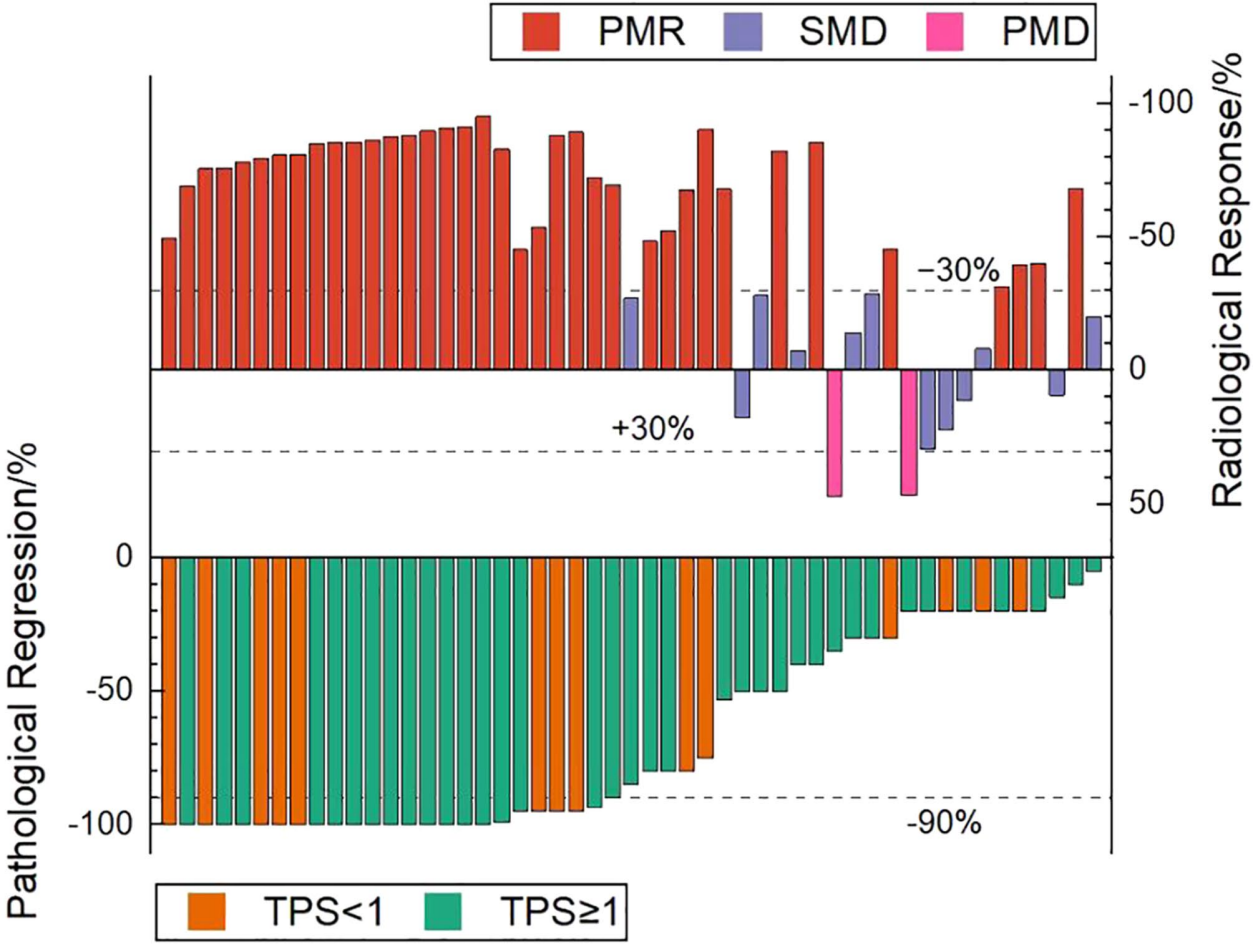
Waterfall plot. Fifty-one patients were evaluated according to PERCIST and the correlation between TPS expression and MPR

**Fig. 2:**
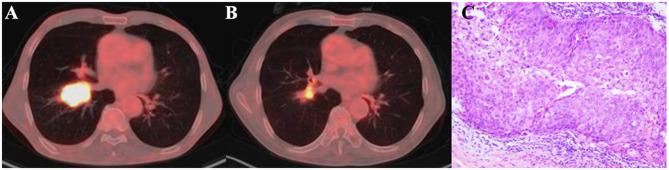
A 73-year-old man with squamous cell lung cancer, who had marked metabolic changes on ^18^F-FDG PET/CT where evaluated as PMR according to PERCIST after three cycles pembrolizumab added to nab-paclitaxel plus cisplatin treatment. (**A**)The scan-1axial fusion image of lung cancer, SULpeak = 10.0. (**B**)The scan-2axial fusion image of lung cancer after neoadjuvant therapy, SULpeak = 5.5; ΔSULpeak% = − 45.2%. (**C**)The surgical pathology showed that MPR was achieved (The residual viable tumor was 5%, less than 10%)

**Fig. 3:**
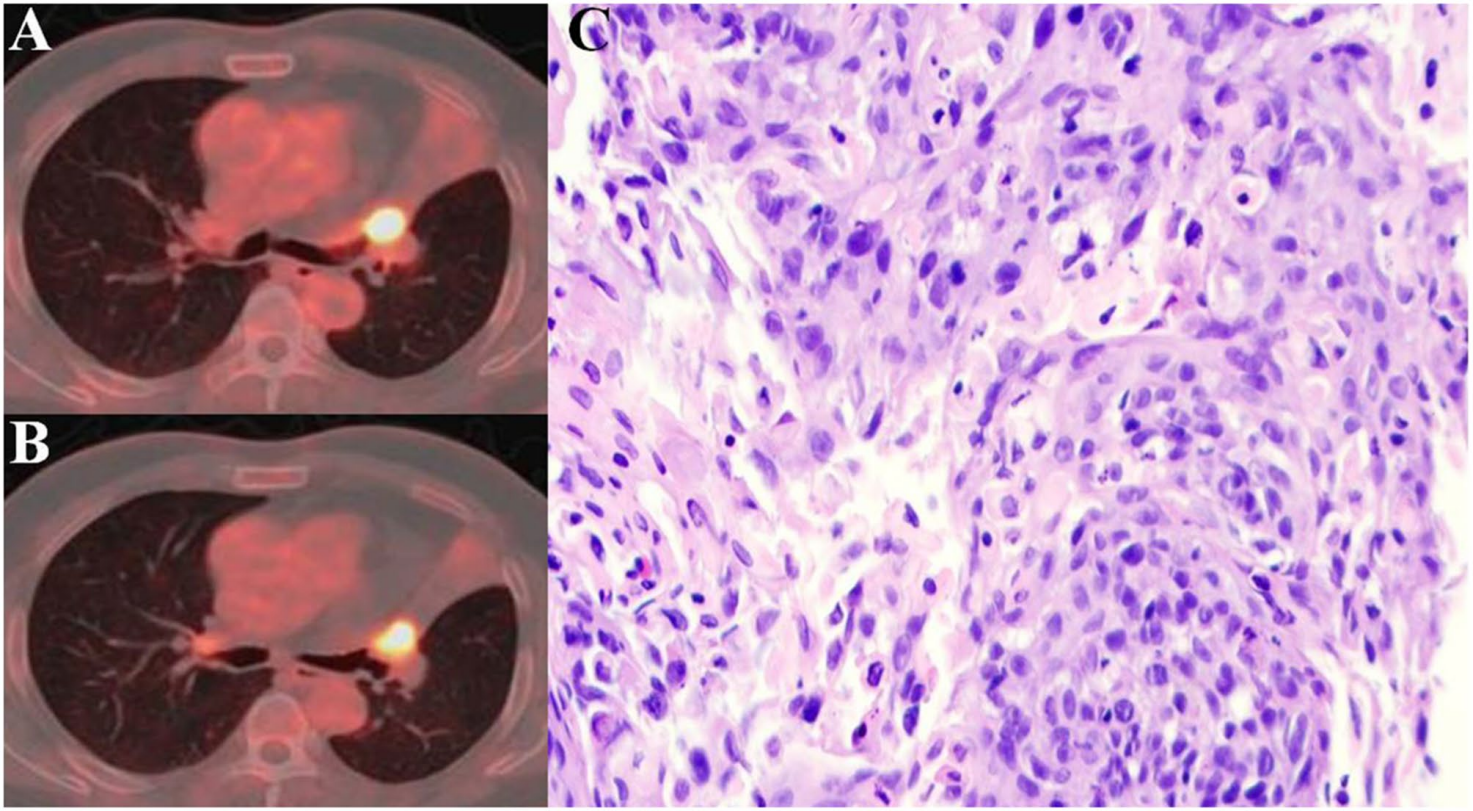
A 54-year-old man with squamous cell lung cancer, who evaluated as SMD according to PERCIST after two cycles toripalimab added to nab-paclitaxel plus cisplatin treatment. (**A**)The scan-1axial fusion image showed high FDG uptake of nodules in the opening of the left upper lobar bronchus. SULpeak = 9.5. (**B**)The scan-2axial fusion image of lung cancer after neoadjuvant therapy, SULpeak = 8.7; ΔSULpeak% = − 8.0%. (**C**)The Resection specimen showed that MPR was not achieved (The residual viable tumor was 80%, more than 10%)

**Fig. 4:**
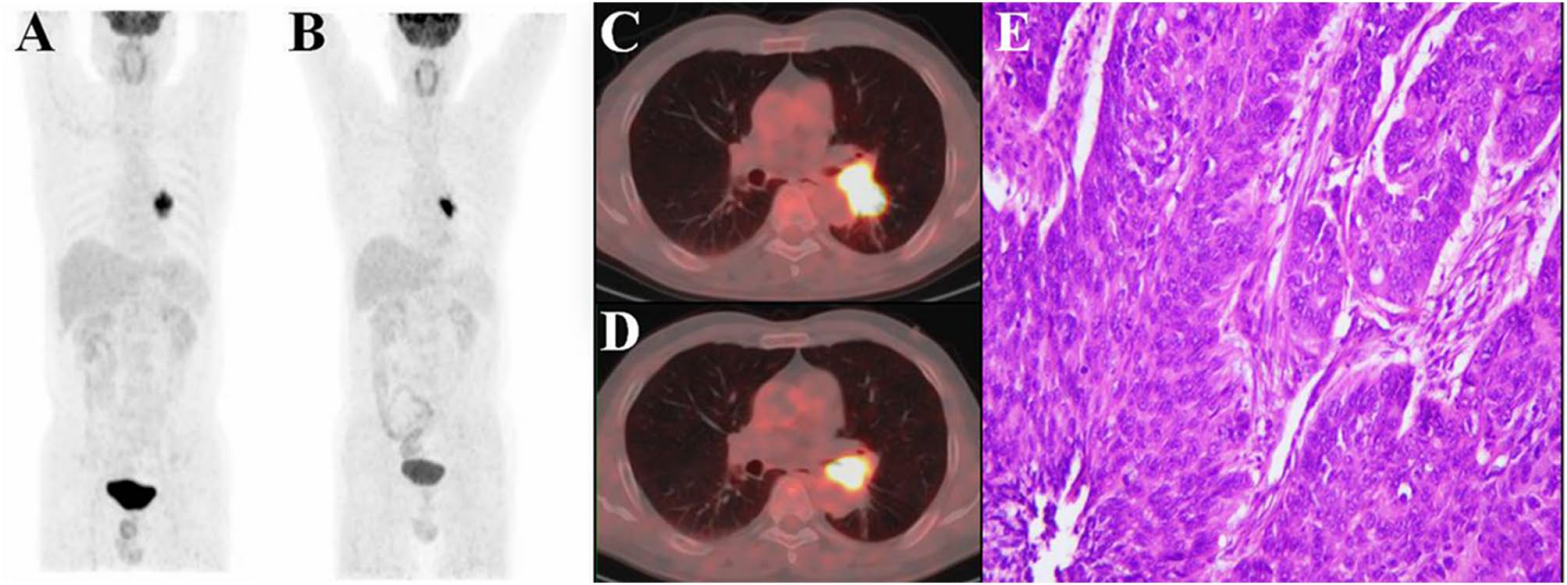
A 53-year-old man with squamous cell lung cancer, who evaluated as PMD according to PERCIST after three cycles toripalimab added to nab-paclitaxel plus cisplatin treatment. (**A**-**B**)MIP (maximum intensity projection) image of scan-1 and scan-2, respectively. (**C**)The scan-1axial fusion image showed high FDG uptake in the left lower lobe near the pulmonary hilum. SULpeak = 10.1; MTV = 25.3; TLG = 150.7. (**D**)The scan-2 axial fusion image showed that both of ΔMTV% and ΔTLG% were decreased(MTV = 15.2,ΔMTV% = −39.9%; TLG = 100.1,ΔTLG% = − 33.6%), but the SULpeak is higher than that of scan-1(SULpeak = 14.8, ΔSULpeak% = 47.1%). (**E**) Resection specimen showed this patient had only 15% of pathological regression

**Fig. 5:**
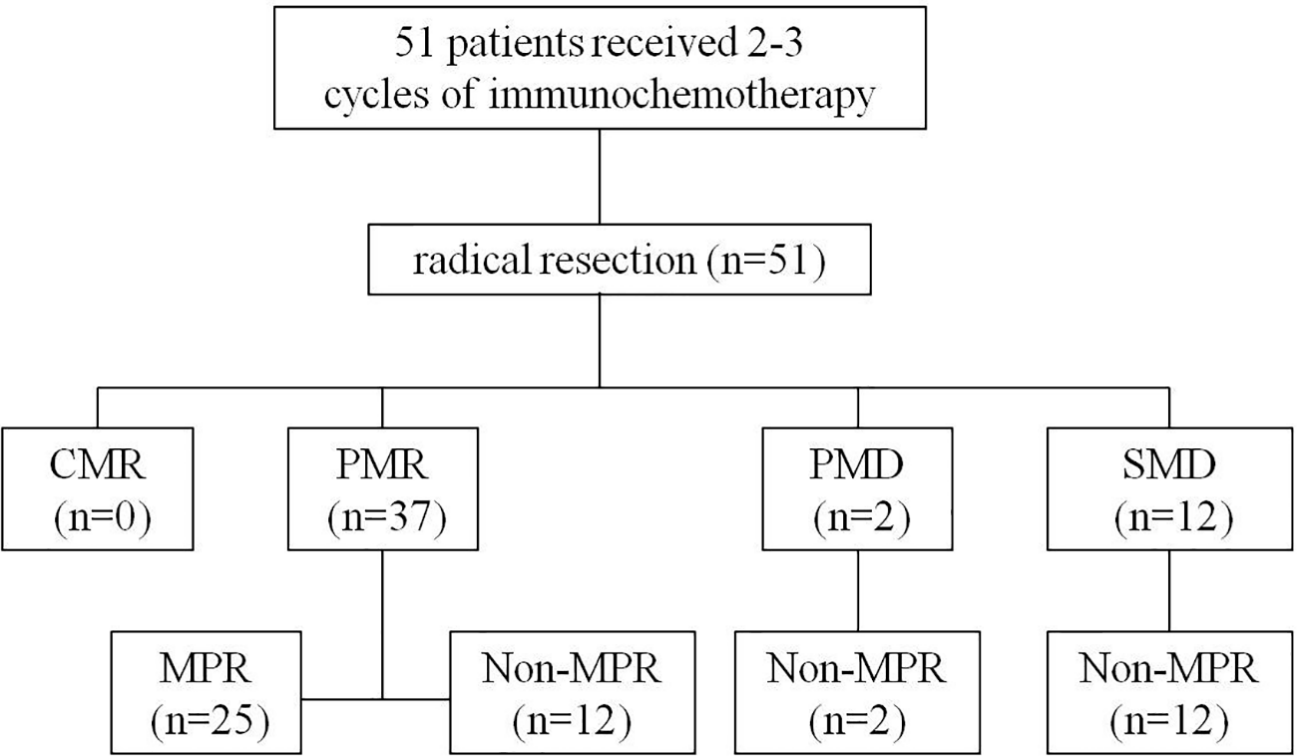
Characteristics of metabolic response according to pathological response

The clinical information and all of metabolic parameters of 25 MPR and 12 non-MPR patients were analyzed. The results showed that SULmax, SULpeak, T/N ratio of scan-2 and ΔSULmax%,ΔSULpeak%, ΔT/N ratio were statistically significant for distinguishing MPR from non-MPR(*p* < 0.001, Table 6). All patients with metabolic SMD and PMD were not acheieved MPR(Figrue 5). Pathology finally revealed that there were 18 patients with no residual lesions (PCR), and the remaining 33 patients had varying degrees of residual lesions.

**Table 6:** Comparison of RECIST 1.1 and PERCIST response assessment

PECIST 1.1	PERCIST				
	CMR	PMR	SMD	PMD	Total
CR	0	0	0	0	0
PR	0	30	5	0	35
SD	0	7	6	2	15
PD	0	0	1	0	1
Total	0	37	12	2	51

The clinical information and all of metabolic parameters of 18 PCR and 33 non-PCR patients were analyzed. The results showed that SULmax, SULpeak, MTV, TLG, T/N ratio of scan-2 and ΔSULmax%, ΔSULpeak%, ΔMTV%, ΔTLG%,ΔT/N ratio were statistically significant for distinguishing PCR from non-PCR(*p* < 0.005, Table 7).

**Table 7:** Values of the metabolic parameters on predicting tumor pathological complete response

Metabolic parameters	Tumor with PCR(*n* = 18)	Tumor without PCR(*n* = 33)	*p* value
Scan-1			
SULmax	11.6 ± 5.8	11.4 ± 4.3	0.921
SULpeak	9.8 ± 5.0	9.9 ± 4.1	0.948
MTV	40.4 ± 53.0	26.7 ± 24.2	0.657
TLG	280.7 ± 432.4	197.5 ± 201.2	0.937
T/N ratio	5.3 ± 2.0	5.5 ± 2.8	0.806
Scan-2			
SULmax	1.9 ± 0.7	7.6 ± 6.8	< 0.001
SULpeak	1.5 ± 0.6	6.2 ± 5.8	< 0.001
MTV	6.2 ± 12.1	10.9 ± 15.8	0.007
TLG	15.2 ± 50.0	72.4 ± 154.8	< 0.001
T/N ratio	0.8 ± 0.3	3.2 ± 3.2	0.003
The percentage changes(Δ%) between scan-1 and scan-2			
ΔSULmax%	-81.0 ± 9.5	-32.5 ± 43.7	< 0.001
ΔSULpeak%	-81.9 ± 10.4	-35.4 ± 40.6	< 0.001
ΔMTV%	-81.3 ± 21.5	-59.3 ± 42.2	0.016
ΔTLG%	-95.2 ± 7.2	-65.4 ± 44.5	< 0.001
ΔT/N ratio%	-80.3 ± 7.4	-40.4 ± 40.3	< 0.001

### Morphologic (RECIST 1.1) vs. metabolic (PERCIST) criteria

In a total of 51 patients, 15 (29.4%) had inconsistent tumor response evaluation, and 36 (70.6%) were consistent. When the PERCIST criteria was adopted, the tumor response of 8(53.3%) patients was upgraded, and that of 7 (46.7%) patients was degraded (Table 6). Of 15 patients with SD, 7 (46.7%) were reclassified in PMR, while 2 were reclassified in PMD by PERCIST criteria. There was no residual tumor in the primary lung cancer of 18 patients after surgery. These patients were all PMR according to the PERCIST criteria, while four patients were SD according to the RECIST 1.1 criteria.The sensitivity, specificity and accuracy of MRP as the gold standard for diagnosis were 100%, 53.8%, 76.5% and 88%,42.3%,64.7% by PERCIST and RECIST 1.1 criteria, with no statistical significance (*P*>0.05).

### Survival results analysis

The median follow-up was 16 months (95%CI, 14 to 19 months). Using univariate cox regression analysis,ΔSULmax%,ΔSULpeak%, ΔMTV%, ΔTLG%,ΔT/N ratio% from primary lung cancer were associated with PFS (Supplementary Table S2). Among the five univariate factors, ΔMTV% had maximum AUC for predicting responders. Patients were divided into ΔMTV%-defined responders and ΔMTV%-defined non-responders according to the cutoff (ΔMTV%<-70% as responders and ΔMTV%≥70% as non-responders). Kaplan meier curves showed ΔMTV%-defined responders demonstrate statistically better PFS than ΔMTV%-defined non-responders(Figrue 6).

**Fig. 6:**
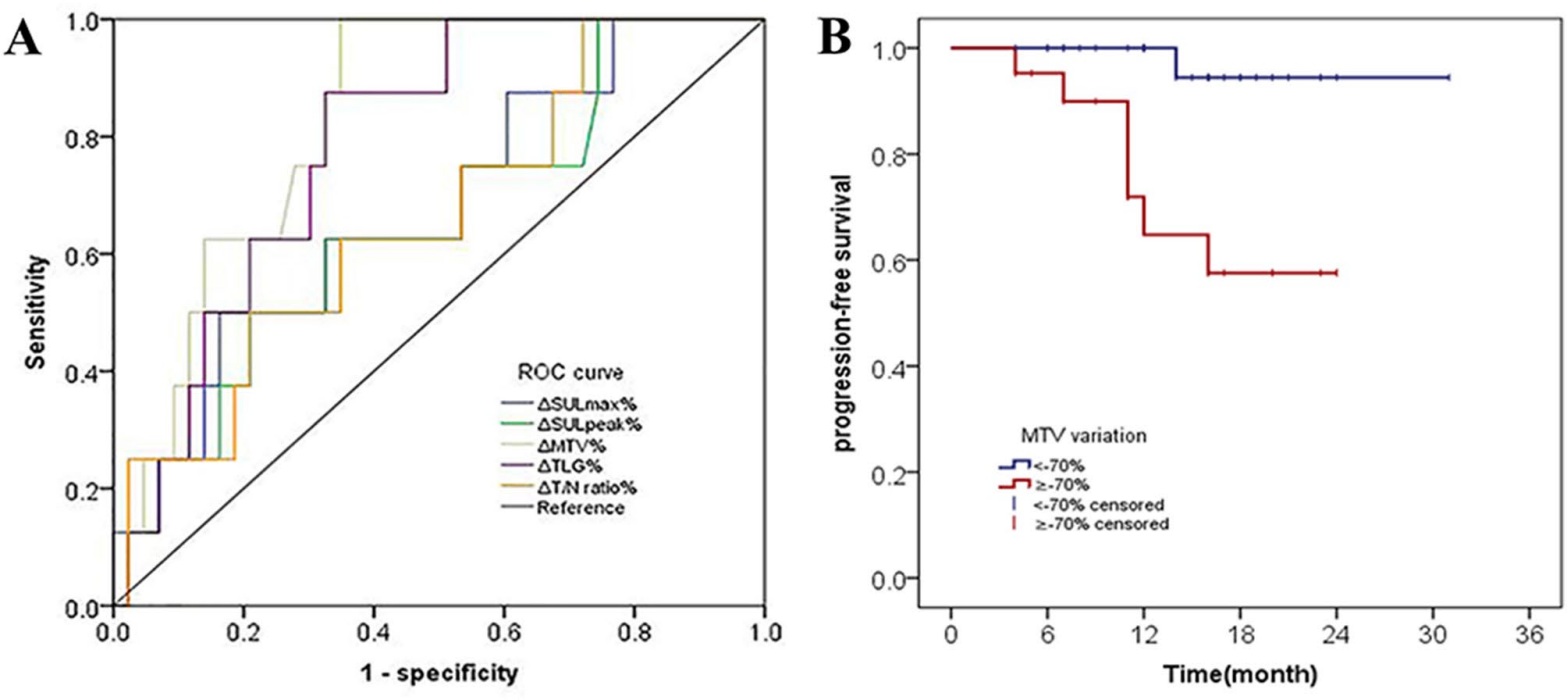
Predictive performance of the metabolic parameters in the identification of prognosis

## Discussion

With the accumulation of convincing evidence, neoadjuvant immunochemotherapy demonstrates remarkable effectiveness in NSCLC without EGFR/ALK alterations, which has been gradually applied in clinical practice. Traditional imaging evaluation criteria are based on morphological changes [17, 18]. Recent clinical trials and retrospective clinical studies have shown no relationship between treatment response assessed by CT images and MPR [15, 19, 20], while ^18^F-FDG PET/CT reflects the changes of tumor metabolism level, which is more suitable for systematic evaluation and monitoring of therapeutic effects [21–23]. This has been demonstrated in both neoadjuvant chemotherapy and immunotherapy for NSCLC [1, 24–27]. The lack of reliable predictive imaging markers to evaluate neoadjuvant immunochemotherapy response is a problem that needs to be solved.To date, little is known about the efficacy of ^18^F-FDG PET/CT to predict response to neoadjuvant immunochemotherapy in patients with resectable NSCLC. In our study, we explored the potential value of ^18^F-FDG PET/CT in predicting MPR of neoadjuvant immunochemotherapy.

MPR is a commonly used alternative predictor of survival in patients with resectable NSCLC receiving neoadjuvant therapy, both in clinical trials and in the real-world [28, 29]. In our retrospective clinical studies, Nearly half of(49.0%,25/51) the patients achieved MPR after neoadjuvant therapy, which is consistent with previous studies [7]. According to the clinical stage, all NSCLC patients with stage I–III received two-three cycles of immunochemotherapy. We observed more squamous-cell carcinoma patients achieving MPR than adenocarcinoma in our study(84% vs. 16%).In the real world, the majority of male smokers with negative driver mutations, so patients with locally advanced squamous cell carcinoma are more likely than patients with adenocarcinoma to receive neoadjuvant immunochemotherapy. The results of our study have more guiding significance for clinical treatment.

There was a statistically significant association between tumor metabolic parameters on ^18^F-FDG PET/CT and the expression of PD1/PD-L1 in resected tumor specimens.Previous studies have shown that changes in metabolic activity are associated with tumor response [30, 31]. How to select the appropriate time point for ^18^F-FDG PET/CT evaluation, and how to evaluate the relationship between the metabolic parameters and the degree of pathological response, these data are worthy of our attention. All patients in our study had some extent degrees of pathological remission after neoadjuvant immunochemotherapy. The scan-2 metabolic parameters and the percentage changes of metabolic parameters of ^18^F-FDG PET/CT after neoadjuvant immunochemotherapy were negatively correlated with the MPR(*p* < 0.05) in our research.Unfortunately, the metabolic parameters of tumor on baseline did not correlate with MPR. Previous studies have shown that ^18^F-FDG PET/CT is an effective methods of monitoring immunotherapy. Our findings suggest that efficacy evaluation should be performed after 2–3 cycles of immunochemotherapy, and SULmax and T/N ratio of scan-2 were the best metabolic parameters to predict MPR. The specificity, sensitivity, and accuracy were 84.6%,88.0%,86.3% and 80.8%,88.0% and 84.3%,respectively.The SULpeak, MTV, TLG of scan-2and all the percentage changes of the metabolic parameters also showed good prediction capabilities to distinguish patients with MPR. Our study showed that ^18^F-FDG PET/CT can help to determine the population who can benefit from neoadjuvant immunochemotherapy followed by radical surgery. Although the PERCIST criteria were evaluated using SULpeak, our study showed that SULmax was more closely associated with MPR.Compared with SULpeak, SULmax reflects the most metabolically active part of the tumor and is easier to measure in patients after neoadjuvant therapy, especially when the residual lesions are small after treatment [16, 32, 33].

According to PERCIST criteria, 37 patients achieved PMR and 25 of them with MPR.All of SMD and PMD patients with non-MPR. Encouragingly, 18 patients’ primary tumor achieved PCR.This result reflects the real situation of NSCLC patients after neoadjuvant immunochemotherapy in clinical practice. We analyzed all of metabolic parameters of these 37 patients and found that SULmax, SULpeak, T/N ratio of scan-2 and ΔSULmax%,ΔSULpeak%, ΔT/N ratio could help distinguish patients with MPR from non-MPR(*p* < 0.001). The lower the SULmax, SULpeak and T/N ratio of scan-2, the easier the MPR of the primary lesion was to achieve after treatment. This result may suggest that preoperative ^18^F-FDG PET/CT is important to evaluate the efficacy of neoadjuvant immunochemotherapy.

We compared PERCIST and RECIST 1.1 criteria to evaluate the response of patients after immunochemotherapy treatment. Of the 15 patients classified as SD according to RECIST 1.1, 6 patients were reclassified as PMR according to PERCIST criteria, 2 patients were reclassified as PMD. More importantly, four patients with no tumor residue were evaluated for PMR by PERCIST and SD by RECIST 1.1. Such changes emphasize that metabolic changes can be detected earlier than morphological changes. SUVmax is the most commonly used metabolic PET/CT parameter, which is based on single pixel value, so cannot well represent the change of FDG uptake distribution in tumor and the overall metabolic state of tumor. Previous studies have proved that volume-based metabolic parameters are significant for the prognosis of primary tumors [34]. Our study demonstrated that ΔSULmax%,ΔSULpeak%, ΔMTV%, ΔTLG%,ΔT/N ratio% from primary lung cancer were associated with PFS, and ΔMTV% was most closely related to PFS. Interestingly, these metabolic parameters were also correlated with patients achieving PCR. Therefore, we recommend measuring ΔMTV% to predict pathological complete response and PFS in NSCLC patients. The metabolic parameter MTV has been used to predict the outcome of NSCLC treatments, including pre-operative [35], chemoradiotherapy [36], chemotherapy [37], and targeted therapy [38].Because there were no death events in all patients, the correlation between OS and metabolic parameters was not studied.

Lopci et al. reported a direct association between the metabolic parameters of ^18^F-FDG PET/CT and the expression of PD-L1 and PD-1 in NSCLC patients [7, 15, 39, 40]. Some studies have shown that even NSCLC patients who have low or negative expression of PD-L1 may benefit from neoadjuvant immunotherapy combined chemotherapy. Our results suggest that patients with tumor PD-L1 expression level of 1% or more than or those with PD-L1 expression level of less than 1% had different degrees of pathological remission. There was no statistical significance between the expression degree of TPS and MPR. These results may suggest that the implementation of neoadjuvant immunochemotherapy may not depend on the expression level of TPS.

Although some studies have reported that neoadjuvant immunotherapy plus chemotherapy has a better effect on NSLCL than chemotherapy alone, there are few reports on ^18^F-FDG PET/CT immunotherapy plus chemotherapy. Previous studies have reported that the antitumor activity of immunotherapy is related to the activation of T cells against cancer cells. Therefore, inflammatory changes secondary to the aggregation of macrophages, neutrophils, and lymphocytes may lead to false positive results. In our patients, there were no false positive results due to changes in immune-related inflammation.In our case, there were 4 patients with reduction in the size of tumor on preoperative ^18^F-FDG PET/CT, but metabolism increased instead. Final pathology confirmed that none of the four patients achieved MPR. This result confirms ^18^F-FDG PET/CT can predict MPR to neoadjuvant immunochemotherapy in resectable non-small cell lung cancer.

Several limitations must be considered in this study. First of all, the retrospective nature of this study may have introduced potential selection and verification biases. Secondly, Most of patients in our study were squamous-cell carcinoma, and only a small proportion were adenocarcinom, which may have biased the results of this study.Thirdly, The observation duration was relatively short after surgery, we did not evaluate clinical endpoints such as disease-free and overall surviva.Hence, large-scale and multicenter study need to be conducted in the future.

## Conclusion

In our study, SULmax and T/N ratio of post-treatment were found to be effective in predicting MPR in NSCLC patients who receiving neoadjuvant immunochemotherapy. Meanwhile, metabolic parameters after treatment were closely related to patients’ PFS, especially ΔTLG%. The metabolic parameters of ^18^F-FDG PET/CT may be a promising method to assess treatment response from neoadjuvant immunochemotherapy before surgical resection.

## Electronic supplementary material

Below is the link to the electronic supplementary material.

Supplementary Material 1

## Data Availability

Data is provided within the manuscript or supplementary information files.

## Abbreviations

^18^F: Fluorine-18
FDG: Fluorodeoxyglucose
PET: Positron emission tomography
CT: Computed tomography
NSCLC: Non-small cell lung cancer
SULmax: Maximum standardized uptake value of lean body mass
SULpeak: Peak standardized uptake value of lean body mass
MTV: Metabolic tumor volume
TLG: Total lesion glycolysis
T/N ratio: Tumor-to- normal liver tissue
ΔSULmax%: (baseline SULmax-post SULmax)/baseline SULmax × 100%
ΔSULpeak%: (baseline SULpeak-post SULpeak)/baseline SULpeak × 100%
ΔMTV%: (baseline MTV-post MTV)/baseline MTV × 100%
ΔTLG%: (baseline TLG-post TLG)/baseline TLG × 100%
ΔT/N ratio%: (baseline T/N-post T/N)/baseline T/*N* × 100%
MPR: Major pathological response
PCR: Pathological complete response
VOI: Volume of Interest
MIP: Maximum intensity projection
CMR: Complete metabolic response
PMR: Partial metabolic response
PMD: Progressive metabolic disease
SMD: Stable metabolic disease
CR: Complete response
PR: Partial response
PD: Progression disease
SD: Stable disease
PFS: Progression-free survival
OS: Overall survival
