# Correction: The utility of ^18^F-FDG PET/CT for predicting the pathological response and prognosis to neoadjuvant immunochemotherapy in resectable non-small-cell lung cancer

**DOI:** 10.1186/s40644-024-00777-6

**Published:** 2024-09-26

**Authors:** Rui Guo, Wanpu Yan, Fei Wang, Hua Su, Xiangxi Meng, Qing Xie, Wei Zhao, Zhi Yang, Nan Li

**Affiliations:** 1 Key Laboratory of Carcinogenesis and Translational Research (Ministry of Education/Beijing), Department of Nuclear Medicine, NMPA Key Laboratory for Research and Evaluation of Radiopharmaceuticals (National Medical Products Administration), Peking University Cancer Hospital & Institute, Beijing, China; 2 Key Laboratory of Carcinogenesis and Translational Research (Ministry of Education/Beijing), Department of Thoracic Surgery I, Peking University Cancer Hospital & Institute, No. 52, Fucheng Road, Haidian District, Beijing, 100142 China; 3 State Key Laboratory of Holistic Integrative Management of Gastrointestinal Cancers, Beijing Key Laboratory of Carcinogenesis and Translational Research, Department of Nuclear Medicine, Peking University Cancer Hospital & Institute, Beijing, 100142 China

**Guo et al. Cancer Imaging (2024) 24:120**

10.1186/s40644-024-00772-x

Following publication of the original article [1], we were made aware that the equal contribution note “Rui Guo and Wanpu Yan contributed equally in this work” is missing, even though present in the submitted manuscript.

The original article has been corrected.
